# Cost-effectiveness of oral food challenge in managing cow's milk allergy under the Brazilian Public Health perspective

**DOI:** 10.1016/j.jped.2026.101625

**Published:** 2026-09-22

**Authors:** Vanessa C.C. Rodrigues, Marcelo C.M. Fonseca, Mauro B. Morais

**Affiliations:** a Universidade Federal de São Paulo, Escola Paulista de Medicina, Post Graduation Program on Nutrition, São Paulo, SP, Brazil; b Universidade Federal de São Paulo, Escola Paulista de Medicina, Department of Gynecology, São Paulo, SP, Brazil; c Universidade Federal de São Paulo, Escola Paulista de Medicina, Department of Pediatrics, Division of Pediatric Gastroenterology, São Paulo, SP, Brazil

**Keywords:** Diagnostic test, Cost-utility, Unified health system, Infant, Meta-analysis

## Abstract

**Objective:**

To analyze the cost-effectiveness and cost-utility of using oral food challenge test (OFC) to manage non-IgE-mediated gastrointestinal cow's milk protein allergy (non-IgE-GI-CMPA) in infants within the Brazilian public health system (SUS).

**Method:**

A decision model was developed to compare two strategies: 1. GUIDELINE-OFC: 100 % of physicians recommending OFC for diagnosis (D-OFC) and evaluation of oral tolerance (OT-OFC), and 2. REAL-WORLD-OFC: OFC performed according to the practices of pediatricians in Brazil (D-OFC indicated by 56 % and 45 % of physicians for mild-to-moderate and severe cases, respectively, and OT-OFC indicated by 93 % of physicians for both cases). The authors assumed that 10 % of parents would refuse the OFC and set the ages of 3.65 and 24 months for model admission and follow-up (time horizon), respectively. Clinical and epidemiological data, primarily from Brazil, data on public health costs, and a 5 % discount rate for both costs and outcomes were used. The effectiveness endpoint was the duration without a cow’s milk elimination diet and quality-adjusted life-year (QALY) values of 0.84 (with allergy) and 0.94 (without allergy).

**Results:**

GUIDELINE-OFC resulted in average savings of USD 241.98, a gain of +0.019 QALYs, and +70 days without a cow’s milk elimination diet per patient. In the probabilistic sensitivity analysis, all cost-effectiveness and 96.5 % of cost-utility simulations demonstrated lower costs and greater effectiveness. Over five years, the public healthcare system would save USD 129 million.

**Conclusions:**

Using OFC in infants with non-IgE-GI-CMPA is a dominant strategy, yielding substantial savings for the SUS and potentially improving nutritional status and quality of life.

## Introduction

Cow's milk protein allergy (CMPA) is the most common food allergy in children [1,2]. About 12 % of parents report adverse milk-related reactions [3]. The prevalence is lower when the oral food challenge test (OFC) is performed to diagnose CMPA, as recommended in guidelines, suggesting overdiagnosis [1,2]. CMPA is a public health issue that can hinder growth, micronutrient intake, feeding, and quality of life [1], [2], [4], [5], [7], [10], [17], [18], [27], [30]. CMPA costs medical treatment, diagnostic testing, drugs, and special diets [5], [6], [14], [15], [28], [29].

CMPA can be IgE-mediated, non-IgE-mediated, or both. Non-IgE CMPA in infants causes nonspecific gastrointestinal symptoms (non-IgE-GI-CMPA). Functional gastrointestinal problems are common in the first six months of life, making differential diagnosis difficult. OFC is the only approved procedure to confirm CMPA diagnosis (D-OFC) [1,2]. At least 50 % of infants on an elimination diet had CMPA ruled out by D-OFC [3], [7], [8], [9]. D-OFC can reduce healthcare costs, prevent unnecessary continued elimination diets, and lessen the restrictive diet's negative impacts on nutrition and quality of life [10], [11], [12], [13] . The OFC should also be used to determine oral tolerance (OT-OFC) in infants on nutritional therapy for at least six months, thereby shortening the elimination diet [1,2].

Two Brazilian studies found that special formulas account for over 90 % of CMPA's expenditures [14], [15]. A recent Brazilian survey revealed that 80 % of pediatricians use special formulas according to guidelines for CMPA infants. However, almost 50 % of pediatricians do not endorse D-OFC [16].

D-OFC and OT-OFC have been recommended for suspected non-IgE-GI-CMPA infants, but no study has evaluated their economic impact. This study aimed to analyze the cost-effectiveness and cost-utility of using OFC to manage non-IgE-GI-CMPA in infants within the Brazilian public health system (*Sistema Único de Saúde*, SUS).

## Methods

### Study design

This pharmacoeconomic study, from the Brazilian SUS perspective, assessed the cost-effectiveness of D-OFC and OT-OFC in the management of infants with suspected non-IgE-GI-CMPA. The public health system approach was chosen because the SUS should provide universal diagnosis and treatment for CMPA in children aged 0 to 24 months in Brazil. A model was developed using data from a survey of 447 pediatricians across the five Brazilian regions on non-IgE-GI-CMPA management [16]. When necessary, clinical and epidemiological data from peer-reviewed scientific publications complemented the pharmacoeconomic model. Study data from Brazil were prioritized. The Federal University of São Paulo Research Ethics Committee approved the study (N.8092060220). A health economic analysis plan was developed prior to conducting the study and is available upon request.

Patient and family perspectives were not directly included in the study design, although the 10 % parental refusal rate for OFC was derived from published literature reflecting real-world parental behavior.

### Pharmacoeconomic model and population

A decision model tree (Supplementary Material 1) was developed to compare two non-IgE-GI-CMPA management strategies:

1. OFC performed as recommended in guidelines (GUIDELINE-OFC), with 100 % of physicians recommending D-OFC and OT-OFC, and
2. OFC performed according to real-world pediatric practice (REAL-WORLD-OFC), reflecting the standard practices of pediatricians in Brazil. D-OFC is recommended by 56 % of physicians for mild-to-moderate cases and 45 % for severe cases; OT-OFC is recommended by 93 % of physicians for both mild-to-moderate and severe cases [16].

Non-exclusively breastfed infants presenting clinical signs of non-IgE-GI-CMPA at an average age of 3.65 months [17], were included in the model. Infants who presented with symptom relief were eligible to perform the D-OFC. For both strategies, a 10 % parental refusal rate to perform the OFCs was assumed [18]. Infants with positive D-OFC plus those whose parents refused to perform the D-OFC remained in the model and were eligible for OT-OFC at 12, 18 and 24 months of age. The exclusion criteria were: (i) infants who failed to present symptom relief on the amino acid-based diagnostic elimination diet; and (ii) infants with a negative D-OFC or OT-OFC (Supplementary Material 1). Both arms used identical costs and clinical and epidemiological data, with the only difference being the frequencies of medical indications for D-OFC and OT-OFC. Table 1 lists time-related parameters [16], [17], [18], probabilities [7], [8], [16], [18], [19], [20], [21], [22], resource use [14], [23], and direct costs [16], [23], [24], [25]. Medication costs were excluded because the most commonly prescribed drugs [16] are mostly symptomatic and were not listed among the pharmaceuticals supplied by SUS for outpatients. Implementation costs (infrastructure, specialized personnel and healthcare service organization) were not considered, since the study adopted the SUS perspective focusing on direct care costs. Supplementary Material 2 provides additional details.

**Table 1:** Clinical and epidemiological data, as well as resource costs, used in constructing the decision model, and the respective values used in the sensitivity analyses, categorized by the severity of clinical manifestations.

Parameters	Baseline value (95% CI: minimum, maximum)		Distribution	Ref.
	Mild to moderate non-IgE-GI-CMPA	Severe non-IgE-GI-CMPA		
**Temporal parameters**				
Age at symptom onset (days)	60 (49, 71)	60 (49, 71)	Log-normal	[18]
Age at model entry (start of elimination diet)	110 (62, 174)	110 (62, 174)	Log-normal	[17]
Time to initial response assessment to diet (days)	14.0 (12.9, 14.2)	14.0 (12.40, 14.70)	Log-normal	[16]
Total duration of diagnostic elimination diet (days)	47.0 (41.0, 53.6)	60.0 (50.7, 68.2)	Log-normal	[16]
Duration of therapeutic elimination diet until OT-OFC (days)	182 (164, 200) a	182 (164, 200) a	Log-normal	[16]
**Probabilities**				
Severe clinical manifestations (see Supplementary Material 2a)	-	0.24 (0.16, 0.36)	Beta	[19]
Referral for specialist evaluation at the initial approach	0.04 (0.02, 0.06)	0.14 (0.11, 0.17)	Beta	[16]
First prescribed formula: extensively hydrolyzed-based formula b	0.85 (0.82, 0.89)	0.35 (0.30, 0.39)	Beta	[16]
Intolerance to extensively hydrolyzed-based formula	0.10 (0.09, 0.11) a	0.40 (0.36, 0.44) a	Beta	[20]
Lack of response to amino acid-based formula	0.05 (0.045, 0.055) a	0.05 (0.045, 0.055) a	Beta	#
In the absence of response to the diet, referral for specialist evaluation	0.22 (0.18, 0.26)	0.51 (0.46, 0.56)	Beta	[16]
Medical indication for performing the D-OFC in REAL-WORLD-OFC	0.56 (0.52, 0.61)	0.45 (0.40, 0.50)	Beta	[16]
Medical indication for performing the D-OFC in GUIDELINE-OFC	1.0 (0.9, 1.0) c	1.0 (0.9, 1.0) c	Beta	[16]
Parental refusal to perform OFCs	0.10 (0.07, 0.15)	0.10 (0.07, 0.15)	Beta	[18]
Performing the D-OFC under medical supervision	0.35 (0.28, 0.40)	0.70 (0.62, 0.77)	Beta	[16]
Hospital admission to perform the D-OFC	0.12 (0.08, 0.16)	0.43 (0.35, 0.51)	Beta	[16]
Positive result of the D-OFC	0.54 (0.44, 0.64)	0.54 (0.44, 0.64)	Beta	[7,8]
Medical indication for performing the OT-OFC in REAL-WORLD-OFC	0.93 (0.91, 0.96)	0.93 (0.92, 0.96)	Beta	[16]
Medical indication for performing the OT-OFC in GUIDELINE-OFC	1.0 (0.9, 1.0) c	1.0 (0.9, 1.0) c	Beta	[16]
Performing the OT-OFC under medical supervision	0.35 (0.32, 0.42)	0.65 (0.59, 0.69)	Beta	[16]
Hospital admission to perform the OT-OFC	0.12 (0.10, 0.17)	0.36 (0.31, 0.41)	Beta	[16]
Positive result of the 1st OT-OFC (at 12 months of age)	0.45 (0.35, 0.55)	0.45 (0.35, 0.55)	Beta	[21,22] #
Positive result of the 2nd OT-OFC (at 18 months of age)	0.26 (0.13, 0.39)	0.26 (0.13, 0.39)	Beta	[22]
**Resource use**				
Monthly formula consumption by age group (cans):				
< 6 months	9.0 (8.1, 9.9) a	9.0 (8.1, 9.9) a	Log-normal	[14]
6 – 12 months	7.5 (6.8, 8.3) a	7.5 (6.8, 8.3) a	Log-normal	[23]
12 < 24 months	6.0 (5.4, 6.6) a	6.0 (5.4, 6.6) a	Log-normal	[23]
Monthly number of medical consultations for symptomatic patients	2	2	Log-normal	[14]
Monthly number of medical consultations for asymptomatic patients	1	1	Log-normal	[14]
**Costs (USD)**				
Extensively hydrolyzed-based formula (can)	11.02 (9.92, 12.13) a	11.02 (9.92, 12.13) a	Log-normal	[23]
Amino acid-based formula (can)	21.01 (18.90, 23.11) a	21.01 (18.90, 23.11) a	Log-normal	[23]
Tests (average cost per patient; see Supplementary Material 2d)	0.32 (0.29, 0.35) a	1.20 (1.08, 1.32) a	Log-normal	[16]
Medical consultations (per visit)	2.00 (1.80, 2.20) a	2.00 (1.80, 2.20) a	Log-normal	[24]
OFC under medical supervision (per test)^d^	9.53 (8.57, 10.48) a	9.53 (8.57, 10.48) a	Log-normal	[23]
Hospitalization for OFC (median cost per patient) e	39.88 (11.07, 54.16)	39.88 (11.07, 54.16)	Log-normal	[25]
**Quality-Adjusted Life Years (QALYs)**				
QALYs with CMPA (during the symptomatic and the elimination diet periods)	0.84 (0.76, 0.92) a	0.84 (0.76, 0.92) a	Beta	[27,23]
QALYs without CMPA (after the elimination diet period)	0.94 (0.85, 1.00) a	0.94 (0.85, 1.00) a	Beta	[27,23]
QALYs without CMPA (prior to the symptomatic period)	1.00 (0.90, 1.00) c	1.00 (0.90, 1.00) c	Beta	
**Discount rate (2nd year)**	0.05 (0.0, 0.10)	0.05 (0.0, 0.10)	Beta	[26]

Abbreviations: CI, confidence interval; CMPA, cow´s milk protein allergy; non-IgE-GI-CMPA, non-IgE gastrointestinal CMPA; OFC, oral food challenge test; D-OFC, diagnostic OFC; OT-OFC, oral tolerance OFC; QALYs, quality-adjusted life years; Ref., reference; GUIDELINE-OFC, OFC performed as recommended in guidelines; REAL-WORLD-OFC, real-world OFC practice.

a A variation of ±10 % was considered to estimate the minimum and maximum values. The maximum value of QALY with CMPA was limited to the value of QALY without CMPA.

b The remaining infants were initially prescribed amino acid-based formula [1,2].

c A variation of −10 % and +0 % was considered to estimate the minimum and maximum values.

d OFC cost performed under medical supervision (in an outpatient clinic), according to components considered in the CONITEC analysis (50 g of infant formula, 6 disposable cups, and labor costs of a doctor, nurse, and nutritionist/hour) [23].

e Hospitalization costs within the SUS for children up to one year of age, due to clinical or diagnostic conditions, with a stay of one day, without the use of the Intensive Care Unit, and discharged as cured or having shown improvement, during the year 2020 (n = 42,070; Supplementary Material 2e).

# Assumption established by the authors (see Supplementary Material 2b).

In the absence of published data on the prevalence of specific severe clinical manifestations of non-IgE-GI-CMPA, anthropometric deficit was chosen as a severity indicator because it is the most objective and measurable outcome available in the literature [1,2]. A comprehensive review and meta-analysis of 387 infants aged 0 to 6 months in seven Brazilian studies found that 24 % (95 % CI = 16 %, 36 %) had anthropometric deficit [19]. This value was adopted as the prevalence of severe clinical manifestations (Supplementary Material 2a).

### Time horizon, discount rate, and currency

The horizon considered was up to 24 months of age, since this is the age range for which the SUS provides hypoallergenic formulas [23]. Given that the timeframe exceeds one year, a discount rate of 5 % was applied to costs and outcomes [26]. The monetary values were reported in US dollars, considering the exchange rate of the Brazilian Real for the year 2023 (1 USD = 4.9949 Brazilian real).

### Cost-effectiveness and cost-utility analyses

OFC's main clinical value lies in its diagnostic accuracy, which can confirm the diagnosis or liberate patients from dietary restrictions if CMPA is ruled out. Therefore, clinical effectiveness was defined as the infant's unrestricted nutrition until 24 months. This period started when the elimination diet was suspended after a negative OFC or removal of suspected CMPA. The cost-effectiveness ratio was determined as the cost per day without the cow's milk elimination diet. The primary effectiveness outcome (time without an elimination diet) was chosen because it is the measure directly related to cost and quality of life in the model. Nevertheless, a positive OFC also provides the relevant clinical value of confirming the diagnosis and guiding appropriate long-term management.

The health states considered in the cost-utility analysis were: (i) with CMPA, during the symptomatic and the elimination diet periods (utility 0.84); and (ii) without CMPA, period after diet discontinuation (utility 0.94). Utility values were derived from the analysis conducted by the National Commission for the Incorporation of Technologies in the SUS (Comissão Nacional de Incorporação de Tecnologias no SUS, CONITEC) [23], which used health-state valuations reported by Protudjer et al. (2015) based on the EQ-5D, a standardized questionnaire for assessing health-related quality of life [27].

### Sensitivity analysis

Univariate and probabilistic sensitivity analyses were performed with 1000 simulations. The clinical and cost variables were adjusted within their respective 95 % confidence intervals (CIs) or, if unavailable, by 10 % in both directions. The discount rate for costs and effectiveness was adjusted within the range of 0 % to 10 %, in line with Brazilian guidelines [26]. The minimum and maximum values of the parameters and the types of distributions used in the sensitivity analyses are detailed in Table 1.

Subgroup analyses were not conducted due to limited data for specific patient subgroups within the Brazilian context.

Distributional effects were not assessed in this study, as the analysis focused on aggregate population-level outcomes from the SUS perspective rather than individual-level impacts.

### Budgetary impact

A five-year budget impact analysis was conducted, assuming that 100 % of Brazilian infants would have access to CMPA diagnosis and treatment through the SUS.

### Supplementary scenario

Because of the uncertainty about the actual frequency of OFCs, additional sensitivity analyses were performed by comparing the GUIDELINE-OFC to the following three scenarios:

1. ONLY-DIAGNOSTIC—OFC: all infants would be indicated for the D-OFC, but none would undergo the OT-OFC;
2. ONLY-TOLERANCE-OFC: no infants would undergo the D-OFC, but all would be indicated for testing the tolerance (OT-OFC) at 12, 18, and 24 months of age, and
3. NONE-OFC: no infant would undergo either D-OFC or OT-OFC, and would remain on an elimination diet until 24 months of age.

As with the GUIDELINE-OFC AND REAL-WORLD-OFC practices, a 10 % parental refusal rate for OFC was considered [18].

### Statistical analysis

All calculations were performed using Microsoft Excel 2019 (Microsoft Corporation, Redmond, Washington, USA).

## Results

### Cost-effectiveness and cost-utility

The GUIDELINE-OFC (D-OFC and OT-OFC in all infants) resulted in average savings of USD 241.98, +0.019 QALYs, and +70 days without a cow’s milk elimination diet per patient (Table 2). Therefore, the routine recommendation of OFC became a dominant strategy, offering lower costs and greater effectiveness. The primary expense was due to the formulas consumed by the infants (Supplementary Material 3A), with about two-thirds of the cost incurred during the therapeutic phase (Supplementary Material 3B). Approximately half of the total expenditure was on extensively hydrolyzed-based formulas, while the other half was on amino acid-based formulas (Supplementary Material 3C). Cost savings were mainly driven by the reduction in the duration of hypoallergenic formula use as demonstrated by both the base-case results (Table 2) and the sensitivity analyses (Supplementary Material 4).

**Table 2:** QALYs, effectiveness (days without a cow’s milk elimination diet), costs, cost-utility and cost-effectiveness according to the frequencies of medical indication to perform the D-OFC and OT-OFC for an age horizon of 24 months.

Strategy	Utility (QALYs)	Effectiveness (Days without a cow's milk elimination diet)	Cost (USD)	Cost/utility (USD/QALY)	Cost/effectiveness (USD/days without a cow’s milk elimination diet)
GUIDELINE-OFC a	1.731	367	945.89	546.45	2.58
REAL-WORLD-OFC	1.712	297	1187.87	693.92	4.00
SUPPLEMENTARY SCENARIOS					
ONLY-DIAGNOSTIC—OFC	1.695	234	1326.54	782.80	5.66
ONLY-TOLERANCE-OFC a	1.695	237	1399.27	825.33	5.90
NONE-OFC	1.633	11	2048.98	1254.45	190.45

Average values per patient included in the model.

Abbreviations: QALYs, quality-adjusted life years; OFC, oral food challenge test; D-OFC, diagnostic OFC; OT-OFC, oral tolerance OFC.

a OT-OFC indicated at 12, 18 and 24 months of age [16]. GUIDELINE-OFC: OFC performed as recommended in guidelines, wherein 100 % of physicians recommend D-OFC and OT-OFC. REAL-WORLD-OFC: management of non-IgE gastrointestinal cow’s milk protein allergy according to real world pediatric practice in Brazil (D-OFC is indicated by 56 % of the physicians for mild to moderate cases and 45 % for severe cases; OT-OFC is indicated by 93 % of the physicians for both cases at 12, 18 and 24 months of age) [16]. ONLY-DIAGNOSTIC—OFC: D-OFC indicated for all infants, but no OT-OFC indication. ONLY-TOLERANCE-OFC: OT-OFC indicated for all infants, but no D-OFC indication. NONE-OFC: no D-OFC or OT-OFC indication - all infants remain on an elimination diet until 24 months of age.

### Sensitivity analysis

Figure 1 presents the results of the probabilistic sensitivity analysis, showing that the GUIDELINE-OFC provides economic, clinical, and quality-of-life benefits. Dominance (lower cost and higher effectiveness) was observed in all cost-effectiveness simulations (Figure 1A) and in 96.5 % of cost-utility simulations (Figure 1B). The univariate sensitivity analysis (Supplementary Material 4) revealed that the results were most sensitive to the age at which the elimination diet was initiated, factors related to formula use (such as formula prices, monthly consumption, and length of the therapeutic diet), and the likelihood of severe clinical manifestations. However, the GUIDELINE-OFC demonstrated lower costs and substantial benefits despite variations across these parameters.

**Fig. 1:**
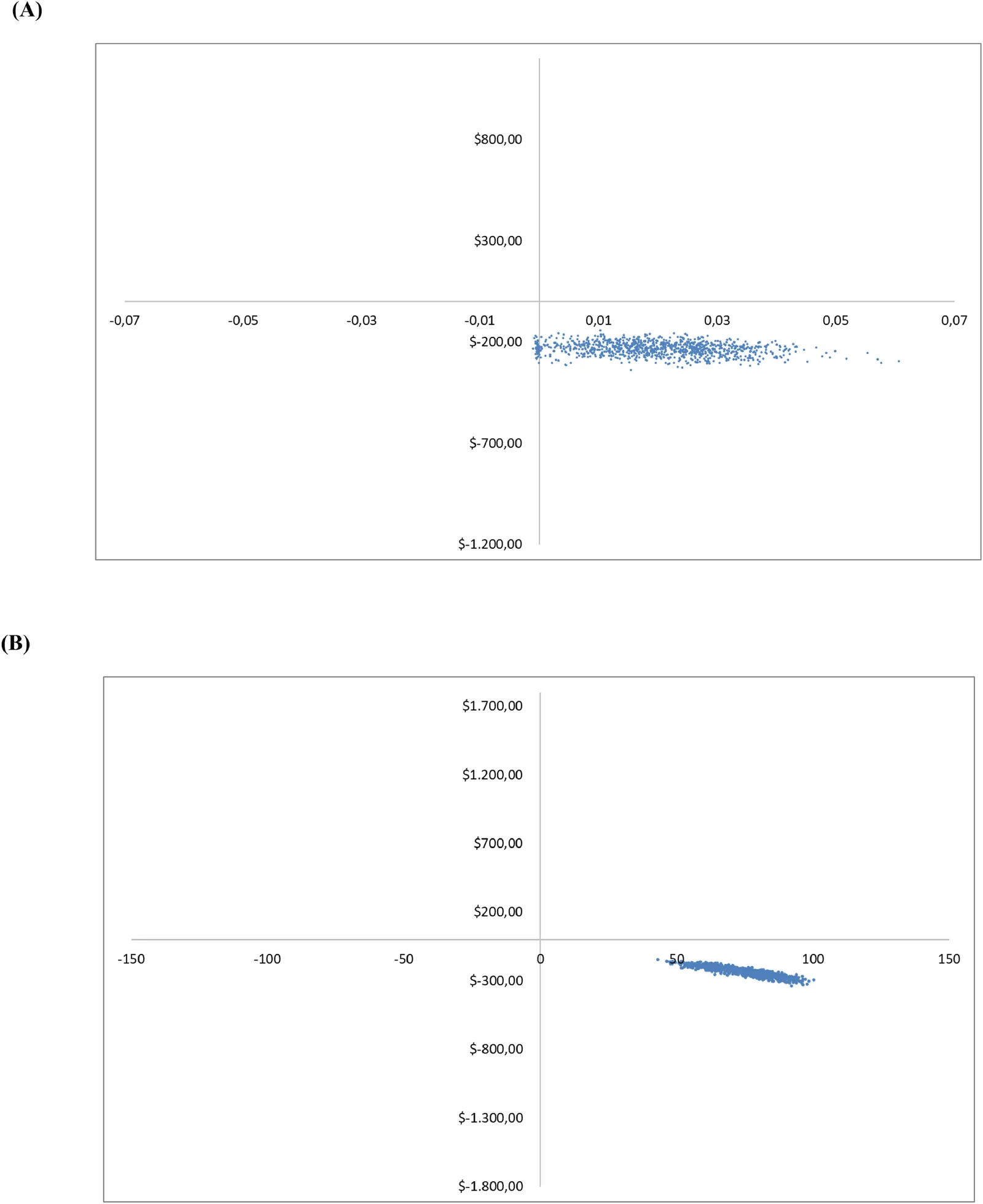
**Probabilistic sensitivity analysis of the incremental cost-utility simulations (A).** The values represent the difference in cost (USD) and utility (QALY) between the GUIDELINE-OFC and the REAL-WORLD-OFC; that is, the savings and the extra years of life in perfect health generated by the GUIDELINE-OFC. **Probabilistic sensitivity analysis of the incremental cost-effectiveness simulations (B).** The values represent the difference in cost (USD) and effectiveness (days without an elimination diet) between the GUIDELINE-OFC and the REAL-WORLD-OFC; that is, the savings and the extra days without an elimination diet generated by the GUIDELINE-OFC. Abbreviations: OFC, oral food challenge test; QALY, quality-adjusted life years; GUIDELINE-OFC, OFC performed as recommended in guidelines; REAL-WORLD-OFC, real-world OFC practice.

### Budget impact

Regarding the budget impact analysis, considering the Brazilian infant population projections, a prevalence of non-IgE-GI-CMPA suspicion of 5.4 % [4], and that 75.48 % of infants with suspected CMPA are formula-fed [17], the OFC recommendation for all Brazilian infants with suspected non-IgE-GI-CMPA resulted in an estimated savings of approximately USD 129 million over five years. These assumptions are detailed in Table 3.

**Table 3:** Budget Impact Analysis of the GUIDELINE-OFC versus the REAL-WORLD-OFC and the SUPPLEMENTARY SCENARIOS over a Five-Year Horizon.

Year	2024	2025	2026	2027	2028	Total
**POPULATION**^a^						
0 < 12 months	2843,515	2815,222	2786,603	2757,920	2729,050	13,932,310
12 < 24 months	-	2840,209	2812,044	2783,552	2754,981	11,190,786
**Population with non-IgE-GI-CMPA**^b^**and formula-fed**^c^						
0 < 12 months	116,391	115,233	114,061	112,887	111,705	570,277
12 < 24 months	-	116,255	115,103	113,936	112,767	458,061
**GUIDELINE-OFC**						
Cost in the 1st year (USD)	86,603,512	85,741,806	84,870,172	83,996,588	86,117,309	424,329,387
Cost in the 2nd year (USD)	-	24,634,833	24,390,541	24,143,413	23,895,599	97,064,386
Cost for the two years (USD)	86,603,512	110,376,639	109,260,713	108,140,001	107,012,908	521,393,774
**REAL-WORLD-OFC**						
Cost in the 1st year (USD)	103,634,381	102,603,218	101,560,173	100,514,796	99,462,604	507,775,171
Cost in the 2nd year (USD)	-	36,311,656	35,951,571	35,587,305	35,222,029	143,072,560
Cost for the two years (USD)	103,634,381	138,914,874	137,511,744	136,102,101	134,684,633	650,847,732
Incremental budget impact (USD)	- 17,030,868	- 28,538,234	- 28,251,031	- 27,962,100	- 27,671,724	- 129,453,958
**SUPPLEMENTARY SCENARIOS**						
**ONLY-DIAGNOSTIC-OFC**						
Cost in the 1st year (USD)	85,929,858	85,074,855	84,210,000	83,343,212	82,470,772	421,028,697
Cost in the 2nd year (USD)	-	71,807,489	71,095,408	70,375,060	69,652,714	282,930,671
Cost for the two years (USD)	85,929,858	156,882,343	155,305,408	153,718,272	152,123,487	703,959,368
Incremental budget impact (USD)	+ 673,654	- 46,505,704	- 46,044,695	- 45,578,271	- 45,110,578	- 182,565,594
**ONLY-TOLERANCE-OFC**						
Cost in the 1st year (USD)	122,778,254	121,556,609	120,320,888	119,082,404	117,835,845	601,574,001
Cost in the 2nd year (USD)	-	42,038,964	41,622,084	41,200,363	40,777,473	165,638,884
Cost for the two years (USD)	122,778,254	163,595,573	161,942,972	160,282,767	158,613,319	767,212,885
Incremental budget impact (USD)	- 36,174,742	- 53,218,934	- 52,682,259	- 52,142,766	- 51,600,410	- 245,819,112
**NONE-OFC**						
Cost in the 1st year (USD)	121,643,219	120,432,868	119,208,571	117,981,536	116,746,501	596,012,694
Cost in the 2nd year (USD)	-	122,538,376	121,323,222	120,093,959	118,861,288	482,816,844
Cost for the two years (USD)	121,643,219	242,971,244	240,531,792	238,075,494	235,607,788	1078,829,538
Incremental budget impact (USD)	- 35,039,707	- 132,594,605	- 131,271,079	- 129,935,493	- 128,594,880	- 557,435,764

Abbreviations: OFC, oral food challenge test; D-OFC, diagnostic OFC; OT-OFC, oral tolerance OFC; non-IgE-GI-CMPA, non-IgE gastrointestinal cow´s milk protein allergy; GUIDELINE-OFC, OFC performed as recommended in guidelines; REAL-WORLD-OFC, real-world OFC practice; ONLY-DIAGNOSTIC—OFC, D-OFC indicated for all infants, but no OT-OFC indication; ONLY-TOLERANCE-OFC, OT-OFC indicated for all infants, but no D-OFC indication; NONE-OFC, no D-OFC or OT-OFC indication.

a Population of Brazilian infants; IBGE/Research Directorate. Coordination of Population and Social Indicators. Department of Demographic Dynamics: Research and Analysis Population Projection of Brazil and Its Federative Units by Sex and Age for the Period 2010–2060 (2018 Edition) [Online Database]. [Cited 2024 Jul 04]. Available from: http://tabnet.datasus.gov.br/cgi/deftohtm.exe?ibge/cnv/projpopuf.def.

b Prevalence of suspected cow's milk protein allergy = 5.4 % [4].

c Frequency of formula-fed infants = 75.8% [17].

### Supplementary scenario

Compared to ONLY-DIAGNOSTIC—OFC, the GUIDELINE-OFC produced an average savings of USD 380.66 per patient, +0.036 QALYs, and +133 days without an elimination diet over a 24-month period (Table 2). Regarding budget impact, savings amounted to USD 182,565,594 over five years (Table 3).

Compared to ONLY-TOLERANCE-OFC, the average savings generated by the GUIDELINE-OFC were USD 453.38 per patient, resulting in +0.036 QALYs and +130 days without an elimination diet during the 24-month period (Table 2). This represents savings of USD 245,819,112 for the SUS over five years (Table 3). In a scenario where 93 % of pediatricians perform the OT-OFC but no D-OFC, the result is close to the ONLY-TOLERANCE-OFC supplementary scenario (cost/utility = $847.83 and cost/effectiveness = $6.38).

The GUIDELINE-OFC, compared to NONE-OFC, yielded an average savings of USD 1103.09 per patient, resulting in +0.098 QALYs and +356 days without an elimination diet within the 24-month period (Table 2). This represents savings of USD 557,435,764 for the SUS over five years (Table 3).

## Discussion

This study demonstrated that the OFC plays a key role in optimizing the use of medical and financial resources for diagnosing and treating non-IgE-GI-CMPA, thereby reducing dietary restrictions and improving quality of life. Recommending OFC for all infants would save USD 241.98, add 0.019 QALYs, and allow 70 extra days without an elimination diet per patient. Even accounting for a 10 % parental refusal rate for OFC, savings would still reach USD 129 million over five years. Furthermore, a positive OFC reduces diagnostic uncertainty, prevents relapses, and supports appropriate long-term management. The effectiveness outcome focused on diet release (negative OFC) captures only one dimension of the overall benefit of OFC.

Two Brazilian studies assessing the cost-effectiveness of CMPA diagnosis and treatment from the SUS perspective used OFC for diagnostic confirmation but did not evaluate different probabilities of performing it [14,15]. As in the current study, formulas accounted for over 95 % of the costs.

Prescription data from the United Kingdom, Norway, and Australia suggest CMPA overdiagnosis. Assuming a 1 % incidence of CMPA, the volume of specialized formula prescribed in 2020 was higher than expected [6]. However, these estimates did not include formula used during diagnostic elimination diets in infants with negative D-OFC results. This omission may be relevant because the diagnostic phase accounted for approximately one-third of formula costs in the present study (Supplementary Material 3b).

Data on the economic impact of OFC are limited. Two U.S. studies found that timely D-OFC would reduce costs and increase QALYs [11,12]. Although consistent with these findings, these studies involved older children with IgE-mediated allergy rather than infants with non-IgE-GI-CMPA. A Dutch study estimated 16 % higher costs if all infants underwent hospital-based double-blind, placebo-controlled OFC rather than open OFC [28]. In the current study, the authors assumed that open OFC would be performed and that only a percentage of patients would undergo the test in a hospital setting, based on frequencies reported in a Brazilian study [16] (Table 1). It should be noted that the most recent guidelines recommend performing open OFC at home for infants with mild-to-moderate non-IgE-GI-CMPA, reserving hospital settings for severe cases [1,2].

The generalizability of the present findings to other healthcare settings should be approached with caution. The magnitude of cost savings may vary depending on local formula pricing, healthcare delivery models, and reimbursement policies.

In March 2022, CONITEC Report No 720 recommended adopting D-OFC and OT-OFC for infants with suspected CMPA within the SUS [23]. This recommendation considered OFC efficacy, safety, cost-effectiveness, and budget impact compared with skin prick and specific IgE tests. The use of D-OFC showed dominance, with lower costs, greater utility, and potential five-year savings of BRL 470–589 million (USD 94–118 million). Although CONITEC also found lower costs and greater benefits, its estimated annual CMPA management cost of BRL 943 (USD 189) was lower than ours, likely due to differences in models and assumptions. Univariate sensitivity analysis (Supplementary Material 4) showed that costs were most sensitive to age at elimination diet initiation, formula-related factors, and severe CMPA frequency. For the same 24-month time horizon, infants entered the CONITEC model at 6 months versus 3.65 months in this study, with infants under 6 months consuming a significantly larger volume of formula. Additionally, the CONITEC study estimated that only 4 % of infants would consume amino acid-based formulas, which have a higher unit cost, while 20 % would consume soy protein-based formulas, which are less costly. Soy protein-based formulas are not recommended for infants with non-IgE-GI-CMPA and were therefore excluded from this study. Finally, the frequency of severe CMPA was not specified in the CONITEC model, a variable that could increase the use of amino acid-based formulas, nor was the OFC parental refusal rate considered.

As the SUS generally provides families with formulas monthly, the model assumes monthly volumes even for use under 30 days. Biweekly dispensing during diagnosis could reduce costs.

The current study did not assess the costs associated with CMPA for families, and no Brazilian studies using this approach were identified. In Bogotá, 17 % of families with infants and children with CMPA spend >15 % of their monthly income on special foods and formulas [5]. In the United States, between 2011 and 2012, families spent an average of US$931 annually on food allergy-related expenses, mainly due to dietary foods [29]. Although in Brazil hypoallergenic formulas are distributed by the SUS for home use, this supply is often delayed [16], which can postpone the start of a nutritionally adequate elimination diet and impact nutritional status. The high prevalence of anthropometric deficits observed in the meta-analysis may partly result from delays in starting an adequate diet, highlighting the importance of prompt diagnosis and access to formulas.

While this study demonstrates economic benefits from the SUS perspective, equity considerations remain important. Delays in formula distribution and limited access to OFC in certain regions may disproportionately affect vulnerable populations, potentially exacerbating health disparities in CMPA management across Brazil. Another source of delay that this study considered is parental refusal of the OFC. Parental refusal, along with events such as acute infections, exacerbations of atopic dermatitis or asthma, and limited appointment availability, are possible delaying factors. It is also important to consider that regional factors, such as food insecurity, difficulties in accessing specialized centers, and geographical and socioeconomic barriers, may raise parental refusal. Policies that ensure equitable assistance must be prioritized.

The authors acknowledge, as a limitation, that anthropometric deficit represents only one of the possible clinical consequences of non-IgE-GI-CMPA and does not fully reflect the severity of the disease itself. Its prevalence was used to define the probability of severe non-IgE-GI-CMPA, which is among the most influential factors on costs, although it did not affect the GUIDELINE-OFC dominance. To address this gap, a meta-analysis estimated the previously unknown rate of any anthropometric deficit among Brazilian infants aged ≤ 6 months with CMPA. Author-provided data were essential for a successful meta-analysis (Supplementary Material 2a). Additionally, the lack of data on the frequency of non-response to the elimination diet is a limitation. Future studies should investigate its prevalence and diagnostic outcomes. The predominant use of Brazilian data strengthens the results’ national relevance.

The projected savings of USD 129 million over five years for the SUS represent a figure of great significance for public policymakers, reinforcing the importance of incorporating OFC into the SUS, in line with the CONITEC recommendation [23]. Moreover, physician adherence may be a barrier as a previous survey revealed that approximately half of Brazilian pediatricians do not routinely recommend D-OFC [16]. The authors recommend continuing medical education programs and the dissemination of these findings to increase adherence to guideline-recommended practices.

In conclusion, the routine use of D-OFC and OT-OFC in infants with non-IgE-GI-CMPA is a dominant strategy, yielding substantial savings for the SUS. The strategy has the potential to improve nutritional status [13] and quality of life, although these outcomes should be considered as potential benefits rather than findings directly demonstrated by this study. Timely access to elimination diets and OFC is necessary, as is the proper inclusion of OFC in the SUS procedure table and its implementation in both outpatient and hospital settings. Physicians should also adhere to OFC recommendations and explain its safety, importance, and benefits to families.

## Institution for indexing in Index Medicus/MEDLINE

Escola Paulista de Medicina, Universidade Federal de São Paulo.

## Funding

This work was supported by the Brazilian National Council for Scientific and Technological Development (CNPq) (grant number 142205/2020-6). The CNPq had no role in the design, analysis, or writing of this article.

## Conflicts of interest

The authors declare no conflict of interest.

## Data Availability

The data that support the findings of this study are available from the corresponding author.

## Appendix Supplementary materials

Image, application 1

Image, application 2

Image, application 3

Image, application 4
