# Supplementary material for: Cost-effectiveness of oral food challenge in managing cow's milk allergy under the Brazilian Public Health perspective
Source: J Pediatr (Rio J). 2026 Sep 22;103(1):101625. doi: 10.1016/j.jped.2026.101625 (PMC13654462; doi:10.1016/j.jped.2026.101625)
Supplement: Supplementary file 1 [file mmc1.docx]

**JPED-D-26-00314_Supplementary Material 1**

**Supplementary Material 1. Decision Tree outlining the steps for diagnosing and treating non-IgE-mediated gastrointestinal cow's milk protein allergy (non-IgE-GI-CMPA). The REAL-WORLD-OFC and the GUIDELINE-OFC, as well as the supplementary scenarios, followed this decision tree, with the only difference being the probabilities of medical indication for D-OFC and OT-OFC.**

**Abbreviations:** AAF, amino acid-based formula; EHF, extensively hydrolyzed-based formula; non-IgE-GI-CMPA, non-IgE-mediated gastrointestinal cow's milk protein allergy; OFC, oral food challenge test; D-OFC, diagnostic OFC; OT-OFC, OFC for assessing the development of tolerance to cow's milk proteins; GUIDELINE-OFC, OFC performed as recommended in guidelines; REAL-WORLD-OFC, real-world OFC practice.
