# Supplementary material for: Cost-effectiveness of oral food challenge in managing cow's milk allergy under the Brazilian Public Health perspective
Source: J Pediatr (Rio J). 2026 Sep 22;103(1):101625. doi: 10.1016/j.jped.2026.101625 (PMC13654462; doi:10.1016/j.jped.2026.101625)
Supplement: Supplementary file 2 [file mmc2.docx]

**JPED-D-26-00314_Supplementary Material 2**

**Supplementary Material 2a. Detailed information on the data used to define the prevalence of severe clinical manifestations of non-IgE-mediated gastrointestinal cow’s milk protein allergy (non-IgE-GI-CMPA).**

Due to the lack of published data on the prevalence of severe clinical manifestations of cow’s milk protein allergy (CMPA), a systematic review and meta-analysis of individual patient data was conducted. The aim was to determine the prevalence of anthropometric deficits at the initial management of CMPA in Brazilian infants who predominantly present with non-IgE-mediated clinical manifestations. Anthropometric deficit was chosen as the outcome because it is typically present in patients with more severe or complex cases, regardless of clinical manifestation [1-3]. The age range from 0 to 6 months was selected based on studies indicating that the onset of CMPA symptoms is predominantly within the first six months of life [4-6].

An anthropometric deficit was defined as a z-score of less than -2.00 standard deviations, according to the 2006 and 2007 World Health Organization reference, for at least one of the following indices: weight-for-age, height-for-age, weight-for-height, or body mass index-for-age. Studies that focused solely on infants who were exclusively breastfed, had IgE-mediated CMPA, and were critically ill were excluded. The systematic review was registered in the International Prospective Register of Systematic Reviews (PROSPERO) (CRD42024508304). The abstract screening tool Rayyan (https://www.rayyan.ai/) was used to screen studies for the systematic review on the prevalence of anthropometric deficits, and the ProMeta software v.3.0 (Internovi, Cesena, Italy) was used to conduct the meta-analysis.

Of the 132 articles identified in the systematic review, 24 were selected, and their authors were contacted to obtain anthropometric data. A total of 387 infants aged 0 to 6 months from seven Brazilian studies were included [6-12]. Individual participant data from each study were analyzed separately. Using the combined data from all studies, a synthesis was conducted with a random-effects meta-analytic model. The meta-analysis found a prevalence of anthropometric deficit of 24% (95% CI = 16% to 36%), which was used to estimate the probability of severe clinical manifestations of non-IgE-GI-CMPA. The limits of the 95% confidence interval were used in the sensitivity analyses.

The results of this systematic review and meta-analysis were presented in 2024 at the 19^th^ Brazilian Congress of Pediatric Gastroenterology and Hepatology, the 5^th^ Brazilian Congress of Pediatric Nutrition, and the 2^nd^ Symposium on Pediatric Nutritional Support [13]. The poster and the abstract published in the conference proceedings are presented below.

**References:**

1. Solé D, Silva LR, Cocco RR, et al. Brazilian Consensus on Food Allergy: 2018 - Part 2 - Diagnosis, Treatment, and Prevention. Joint position paper of the Brazilian Society of Pediatrics and the Brazilian Association of Allergy and Immunology. Arq Asma Alerg Imunol 2018;2:39-82. doi.org/10.5935/2526-5393.20180005.
2. Toca MC, Morais MB, Vázquez-Frias R, Becker-Cuevas DJ, Boggio-Marzet CG, Delgado-Carbajal L, et al; the Food Allergy Working Group of the Latin American Society for Pediatric Gastroenterology, Hepatology and Nutrition. Consensus on the diagnosis and treatment of cow's milk protein allergy of the Latin American Society for Pediatric Gastroenterology, Hepatology and Nutrition. Rev Gastroenterol Mex (Engl Ed). 2022 Apr-Jun;87(2):235-250. doi: 10.1016/j.rgmxen.2022.01.002. Epub 2022 May 24.
3. Vandenplas Y, Broekaert I, Domellöf M, Indrio F, Lapillonne A, Pienar C, et al. An ESPGHAN Position Paper on the Diagnosis, Management, and Prevention of Cow's Milk Allergy. J Pediatr Gastroenterol Nutr. 2024 Feb;78(2):386-413. doi: 10.1097/MPG.0000000000003897. Epub 2023 Jul 26.
4. Aguiar AL, Maranhão CM, Spinelli LC, de Figueiredo RM, Maia JM, Gomes RC, et al. Clinical and follow up assessment of children in a program directed at the use of formulas for cow's milk protein allergy. Rev Paul Pediatr. 2013 Jun;31(2):152-8. English, Portuguese. doi: 10.1590/s0103-05822013000200004.
5. Taveira GR, Fernandes CDR, Silva YFR, de Aquino MCB, da Silva ACMV, de Faria CP, et al. Evolution of nutritional status and associated factors among formula-fed infants with cow's milk protein allergy in a government program. Arch Public Health. 2023 May 12;81(1):90. doi: 10.1186/s13690-023-01094-3.
6. Jardim-Botelho A, Martins TG, Motta-Franco J, Meyer R, Fontes Vieira SC, Protásio BF, et al. Growth and Nutritional Biomarkers in Brazilian Infants with Cow's Milk Allergy at Diagnosis and 18-Month Follow-Up: A Prospective Cohort Study. Pediatr Gastroenterol Hepatol Nutr. 2023 Nov;26(6):355-369. doi: 10.5223/pghn.2023.26.6.355. Epub 2023 Oct 30.
7. Vieira MC, Morais MB, Spolidoro JV, Toporovski MS, Cardoso AL, Araujo GT, et al. A survey on clinical presentation and nutritional status of infants with suspected cow' milk allergy. BMC Pediatr. 2010 Apr 23;10:25. doi: 10.1186/1471-2431-10-25.
8. Alves JQN, Mendes JFR, Jaborandy M de L. Perfil nutricional e consumo dietético de crianças alérgicas à proteína do leite de vaca acompanhadas em um hospital infantil de Brasília/DF, Brasil [Nutritional profile and dietary intake of children allergic to cow’s milk protein accompanied by a children’s hospital, in Brasilia, Brazil]. Com Ciênc Saúde. 2017;28:402-12. Portuguese.
9. Meyer R, Wright K, Vieira MC, Chong KW, Chatchatee P, Vlieg-Boerstra BJ, et al. International survey on growth indices and impacting factors in children with food allergies. J Hum Nutr Diet. 2019 Apr;32(2):175-184. doi: 10.1111/jhn.12610. Epub 2018 Nov 9.
10. Assis PP, Menezes JSS, Diniz AS, Antunes MMC, Cabral PC. Growth of infants with gastrointestinal manifestations of cow’s milk protein allergy. Rev Nutr 2022;35:e210075. doi: 10.1590/1678-9865202235e210075.
11. Frizzo J, Rodrigues VCC, Speridião PGL, Morais MB. Evaluation of the complementary feeding practices, dietary intake, and nutritional status of infants on a cow's milk protein elimination diet. J Pediatr (Rio J). 2022 May-Jun;98(3):256-263. doi: 10.1016/j.jped.2021.06.005. Epub 2021 Jul 22.
12. de Faria DPB, Sillos MD, Speridião PDGL, de Morais MB. Outcome of food intake and nutritional status after discontinuation of a cow's-milk-free diet post negative oral food challenge in infants and children. Allergol Immunopathol (Madr). 2022 Jan 1;50(1):1-8. doi: 10.15586/aei.v50i1.471.
13. Rodrigues VCC, Fonseca MCM, Morais MB. Déficit antropométrico em lactentes com alergia ao leite de vaca no Brasil: revisão sistemática e metanálise de prevalência utilizando dados individuais. In: Anais do 19º Congresso Brasileiro de Gastroenterologia e Hepatologia Pediátricas; 5º Congresso Brasileiro de Nutrologia Pediátrica; 2ª Simpósio de Suporte Nutricional Pediátrico; 2024. [Cited 2025 Jan 22]. Available from: http://anais.sbp.com.br/trabalhos-de-congressos-da-sbp/19-congresso-brasileiro-de-gastroenterologia-e-hepatologia-pediatricas/0129-deficit-antropometrico-em-lactentes-com-alergia-ao.pdf

**Supplementary material 2b. Detailed information on estimates of the prevalence of other clinical variables that were not found in the literature.**

The positivity rate of the oral food challenge test for assessing the development of oral tolerance (OT-OFC) in 12-month-old infants was estimated at 45%, using the equation proposed by Gidwani and Russell [1] and assuming a positivity rate of 26% (11/43) at 18 months [2].

Clinical studies did not report the prevalence of failure to feed amino acid-based formula in infants with suspected CMPA; therefore, the authors estimated this prevalence at 5%.

**References:**

1. Gidwani R, Russell LB. Estimating Transition Probabilities from Published Evidence: A Tutorial for Decision Modelers. Pharmacoeconomics. 2020 Nov;38(11):1153-1164. doi: 10.1007/s40273-020-00937-z. Erratum in: Pharmacoeconomics. 2020 Nov;38(11):1277. doi: 10.1007/s40273-020-00958-8.
2. Saarinen KM, Pelkonen AS, Mäkelä MJ, Savilahti E. Clinical course and prognosis of cow's milk allergy are dependent on milk-specific IgE status. J Allergy Clin Immunol. 2005 Oct;116(4):869-75. doi: 10.1016/j.jaci.2005.06.018. Epub 2005 Aug 19.

**Supplementary material 2c. Detailed information on the prices for formulas, oral food challenge test (OFC), and procedures.**

We considered the prices and quantities of hypoallergenic formulas and OFC listed in Recommendation Report No. 720/2022 [1], as well as the prices listed in the SUS Management System Table for procedures, medications, orthotics, prosthetics, and special materials [2]. These prices are presented in Table 1.

**References:**

1. Brazil Ministry of Health. Secretariat of Science, Technology, Innovation, and Strategic Inputs in Health. National Commission for the Incorporation of Technologies in the Unified Health System – CONITEC. Recommendation Report No. 720. Oral food challenge for cow's milk protein allergy. Brasília, DF, March/2022. [Cited 2024 Mar 15]. Available from: https://www.gov.br/conitec/pt-br/midias/relatorios/2022/20220401_relatorio_720_tpo_aplv.pdf
2. Brazil Ministry of Health. Datasus. SIGTAP - System for the Management of the Table of Procedures, Medications, and OPM of SUS. [Cited 2024 Mar 19]. Available from: http://sigtap.datasus.gov.br/tabela-unificada/app/sec/inicio.jsp

**Supplementary material 2d. Estimated costs of tests requested in the initial management of infants with suspected non-IgE-GI-CMPA according to the severity of clinical manifestations. The average cost per patient was estimated using the frequency of test requests reported in a national study.^1^**

| Lab tests | Mild to moderate clinical manifestations of non-IgE-GI-CMPA (n = 446) | | | Severe clinical manifestations of non-IgE-GI-CMPA (n = 422) | | |
| --- | --- | --- | --- | --- | --- | --- |
|  | N | % | Unit cost^46^ (R$) | N | % | Unit cost^46^ (R$) |
| Specific IgE for cow’s milk proteins or fractions | 26 | 5.8% | 9.25 | 48 | 11.4% | 9.25 |
| Total IgE | 5 | 1.1% | 9.25 |  |  |  |
| Soy-specific IgE |  |  |  | 1 | 0.2% | 9.25 |
| Prick test |  |  | 1.77 | 3 | 0.7% | 1.77 |
| Complete blood Count | 21 | 4.7% | 4.11 | 84 | 19.9% | 4.11 |
| Reticulocyte Count | 1 | 0.2% | 2.73 |  |  |  |
| Coagulogram | 1 | 0.2% | 2.73 | 1 | 0.2% | 2.73 |
| Blood culture |  |  |  | 1 | 0.2% | 11.49 |
| Ferritin level | 5 | 1.1% | 15.59 | 23 | 5.5% | 15.59 |
| Serum iron, transferrin level |  |  |  | 9 | 2.1% | 7.63 |
| Fecal occult blood Count | 13 | 2.9% | 1.65 | 16 | 3.8% | 1.65 |
| Fecal alpha-1-antitrypsin level | 4 | 0.9% | 3.68 | 4 | 0.9% | 3.68 |
| Coprological, coprogram, protoparasitological feces, fecal leukocytes, fecal pH. RS tests | 10 | 2.2% | 6.34 | 10 | 2.4% | 6.34 |
| Stool culture and viral and bacterial enteropathogens | 3 | 0.7% | 15.87 | 7 | 1.7% | 15.87 |
| Liver function | 1 | 0.2% | 4.02 | 9 | 2.1% | 4.02 |
| Renal function | 1 | 0.2% | 3.70 | 8 | 1.9% | 3.70 |
| Glycemia |  |  |  | 1 | 0.2% | 1.85 |
| Electrolytes. blood gas analysis |  |  |  | 10 | 2.4% | 6.48 |
| T4, TSH |  |  | 17.72 | 1 | 0.2% | 17.72 |
| Total protein and protein fractions | 1 | 0.2% | 1.85 | 5 | 1.2% | 1.85 |
| C-reactive protein | 3 | 0.7% | 9.25 | 16 | 3.8% | 9.25 |
| Urine 1 and urine culture | 4 | 0.9% | 9.32 | 29 | 6.9% | 9.32 |
| Anti-endomysium and anti-transglutaminase antibodies |  |  |  | 1 | 0.2% | 18.55 |
| Vitamins D. and B12 tests |  |  |  | 7 | 1.7% | 30.48 |
| Abdominal ultrasound | 1 | 0.2% | 37.95 | 5 | 1.2% | 37.95 |
| Echocardiogram |  |  |  | 1 | 0.2% | 67.86 |
| Average cost per patient ^a^ |  |  | 1.61 |  |  | 5.99 |

^a^ Average cost per patient, assuming that each one is seen by a physician [1].

Abbreviations: Non-immunoglobulin E-mediated gastrointestinal cow's milk protein allergy (non-IgE-GI-CMPA).

Values are presented in Brazilian Real. Considering the average exchange rate in 2023 (USD 1.00 = R$ 4.9949), the average costs of the tests requested per patient with mild-to-moderate and severe clinical manifestations of non-IgE-GI-CMPA were USD 0.3223 and USD 1.1992, respectively.

**Reference:** 1. Rodrigues VCC, Fonseca MCM, Solé D, Sanudo A, Morais MB. Real-life management of gastrointestinal cow's milk protein allergy in Brazilian infants. Allergol Immunopathol (Madr). 2025 Jul 1;53(4):68-77. doi: 10.15586/aei.v53i4.1323.

**Supplementary material 2e: Estimated hospitalization costs for performing the oral food challenge test**

The values are presented as the mean, median, and 95% confidence interval of hospitalization costs within the Brazilian Unified Health System for children up to one year of age, due to clinical or diagnostic conditions, with a stay of one day, without the use of the Intensive Care Unit, and discharged as cured or having shown improvement, during the year 2020 (n=42,070) [1].

Values are presented in Brazilian Real. Considering the average exchange rate in 2023 (USD 1.00 = R$ 4.9949), the average cost of hospitalization was USD 39.8767 (95% CI 11.0653; 54.1632).

**Reference:** 1. Brazil Ministry of Health. Datasus. Database of the Hospital Information System (SIH) of the Unified Health System (SUS). [Cited 2024 Mar 28]. Available from: http://sihd.datasus.gov.br/principal/index.php.
