# Supplementary material for: Cost-effectiveness of oral food challenge in managing cow's milk allergy under the Brazilian Public Health perspective
Source: J Pediatr (Rio J). 2026 Sep 22;103(1):101625. doi: 10.1016/j.jped.2026.101625 (PMC13654462; doi:10.1016/j.jped.2026.101625)
Supplement: Supplementary file 3 [file mmc3.docx]

**Supplementary Material 3a.** Contribution of medical consultations and tests, oral food challenge tests and hypoallergenic formulas to total costs, according to the type of practice (average values in US dolars per patient included in the model).

**Supplementary Material 3b.** Costs associated with hypoallergenic formulas during the initial phase of the evaluation of the response to the elimination diet and confirmation of the diagnosis (diagnostic elimination diet), as well as during the treatment phase (therapeutic elimination diet), according to the type of practice (average costs in US dollars per patient included in the model).

**Supplementary Material 3c.** Total costs associated with the use of extensively hydrolyzed-based and free amino acid-based formulas, according to the type of practice (average costs in US dollars per patient included in the model).
