# Supplementary material for: Cost-effectiveness of oral food challenge in managing cow's milk allergy under the Brazilian Public Health perspective
Source: J Pediatr (Rio J). 2026 Sep 22;103(1):101625. doi: 10.1016/j.jped.2026.101625 (PMC13654462; doi:10.1016/j.jped.2026.101625)
Supplement: Supplementary file 4 [file mmc4.docx]

**JPED-D-26-00314_Supplementary Material 4**

**Supplementary material 4.** Univariate sensitivity analysis

|  | **Input Value (Deterministic)** | **Minimum Value** | **Maximum Value** | **Cost-Utility for Minimum Value (USD/QALY)** | **Cost-Utility for Maximum Value (USD/QALY)** | **Cost-effectiveness for the minimum value (USD/days without a cow’s milk elimination diet)** | **Cost-effectiveness for the maximum value (USD/days without a cow’s milk elimination diet)** |
| --- | --- | --- | --- | --- | --- | --- | --- |
| **Unit costs (USD)** |  |  |  |  |  |  |  |
| Medical consultations (per visit) | 2.00 | 1.80 | 2.20 | -12619.78 | -12664.54 | -3.46 | -3.47 |
| Exams (mild to moderate non-IgE-GI-CMPA. average cost per patient) | 0.32 | 0.29 | 0.35 | -12642.16 | -12642.16 | -3.46 | -3.46 |
| Exams (severe non-IgE-GI-CMPA, average cost per patient) | 1.20 | 1.08 | 1.32 | -12642.16 | -12642.16 | -3.46 | -3.46 |
| EHF (per can) | 11.02 | 9.92 | 12.13 | -12052.25 | -13232.07 | -3.30 | -3.63 |
| AAF (per can) | 21.01 | 18.91 | 23.11 | -11977.51 | -13306.80 | -3.28 | -3.65 |
| OFC (per test) | 9.53 | 8.57 | 10.48 | -12646.26 | -12638.06 | -3.47 | -3.46 |
| Hospitalization for the OFC procedure (cost per patient) | 39.88 | 11.07 | 54.16 | -12704.45 | -12611.27 | -3.48 | -3.46 |
| **Probabilities and outcomes applied to the management of both mild to moderate and severe non-IgE-GI-CMPA** | | | |  |  |  |  |
| Severe non-IgE-GI-CMPA | 0.24 | 0.16 | 0.36 | -12206.08 | -13277.98 | -3.34 | -3.64 |
| Age at symptom onset (days) | 60.00 | 49.00 | 71.00 | -12642.16 | -12642.16 | -3.46 | -3.46 |
| Age at which the elimination diet was initiated (days) | 110.00 | 62.40 | 174.00 | -11197.89 | -15294.42 | -3.07 | -4.19 |
| Number of formula cans (0-6 months old) (units) | 9.00 | 8.10 | 9.90 | -12528.50 | -12755.81 | -3.43 | -3.50 |
| Number of formula cans (6–12 months old) (units) | 7.50 | 6.75 | 8.25 | -11982.79 | -13301.53 | -3.28 | -3.64 |
| Number of formula cans (12–24 months old) (units) | 6.00 | 5.40 | 6.60 | -12160.63 | -13123.69 | -3.33 | -3.60 |
| Duration of the first therapeutic diet (6–12 months old) (days) | 182.00 | 163.80 | 200.20 | -13297.94 | -12048.01 | -3.64 | -3.30 |
| Duration of the second therapeutic diet (12–18 months old) (days) | 182.00 | 163.80 | 200.20 | -13037.05 | -12270.48 | -3.57 | -3.36 |
| Duration of the third therapeutic diet (18–24 months old) (days) | 183.00 | 164.70 | 201.30 | -12834.30 | -12455.69 | -3.52 | -3.41 |
| Parental refusal for OFC procedure | 0.10 | 0.07 | 0.15 | -12686.80 | -12579.22 | -3.48 | -3.45 |
| Diagnostic OFC (D-OFC) positivity rate | 0.54 | 0.44 | 0.64 | -12763.62 | -12471.36 | -3.50 | -3.42 |
| Oral tolerance OFC (OT-OFC) positivity rate at 12 months old | 0.45 | 0.35 | 0.55 | -12721.94 | -12572.62 | -3.49 | -3.45 |
| OT-OFC positivity rate at 18 months old | 0.26 | 0.13 | 0.39 | -12688.85 | -12600.69 | -3.48 | -3.45 |
| QALYs with allergy | 0.84 | 0.76 | 0.92 | -6870.74 | -79013.49 | -3.46 | -3.46 |
| QALYs without allergy | 0.94 | 0.85 | 1.00 | -210702.63 | -7901.35 | -3.46 | -3.46 |
| QALY before symptoms (without allergy) | 1.00 | 0.90 | 1.00 | -12642.16 | -12642.16 | -3.46 | -3.46 |
| Discount rate applied to costs and outcomes in the second year | 0.00 | 0.00 | 0.10 | -12606.74 | -12675.78 | -3.45 | -3.47 |
| **Probabilities and outcomes applied only to the management of mild to moderate non-IgE-GI-CMPA** | | |  |  |  |  |  |
| Upon suspecting CMPA, refer for specialist consultation | 0.04 | 0.02 | 0.06 | -12642.16 | -12642.16 | -3.46 | -3.46 |
| Prescription of EHF | 0.85 | 0.82 | 0.89 | -12819.09 | -12441.99 | -3.51 | -3.41 |
| Intolerance to EHF | 0.10 | 0.09 | 0.11 | -12598.72 | -12685.65 | -3.45 | -3.48 |
| Non-response to AAF | 0.05 | 0.05 | 0.06 | -12653.29 | -12630.98 | -3.47 | -3.46 |
| Refer patient with formula intolerance for specialist consultation | 0.22 | 0.18 | 0.26 | -12642.46 | -12641.88 | -3.46 | -3.46 |
| Supplemental Digital Content 4 (continuation) |  |  |  |  |  |  |  |
| **Probabilities and outcomes applied only to the management of mild to moderate non-IgE-GI-CMPA** | | |  |  |  |  |  |
| Time to initial response assessment to diet (days) | 13.60 | 12.90 | 14.20 | -12640.74 | -12643.37 | -3.46 | -3.46 |
| Total duration of the diagnostic diet (days) | 47.30 | 41.00 | 53.60 | -12488.09 | -12800.08 | -3.42 | -3.51 |
| Medical indication for performing the D-OFC in REAL-WORLD-OFC PRACTICE | 0.56 | 0.52 | 0.61 | -12571.59 | -12733.66 | -3.44 | -3.49 |
| Medical indication for performing the D-OFC in GUIDELINE-OFC PRACTICE | 1.00 | 0.90 | 1.00 | -12822.26 | -12642.16 | -3.51 | -3.46 |
| D-OFC under medical supervision | 0.35 | 0.28 | 0.40 | -12653.04 | -12635.00 | -3.47 | -3.46 |
| Hospitalization for conducting the D-OFC | 0.12 | 0.08 | 0.16 | -12642.16 | -12642.16 | -3.46 | -3.46 |
| Medical recommendation for conducting the OT-OFC in REAL-WORLD-OFC PRACTICE | 0.93 | 0.91 | 0.96 | -12550.74 | -12762.10 | -3.44 | -3.50 |
| Medical recommendation for conducting the OT-OFC in GUIDELINE-OFC PRACTICE | 1.00 | 0.90 | 1.00 | -13017.77 | -12642.16 | -3.57 | -3.46 |
| OT-OFC under medical supervision | 0.35 | 0.32 | 0.42 | -12639.57 | -12647.85 | -3.46 | -3.47 |
| Hospitalization for conducting the OT-OFC | 0.12 | 0.10 | 0.17 | -12646.20 | -12628.41 | -3.47 | -3.46 |
| **Probabilities and outcomes applied only to the management of severe non-IgE-GI-CMPA** | |  |  |  |  |  |  |
| Upon suspecting CMPA, refer for specialist consultation | 0.14 | 0.11 | 0.17 | -12642.16 | -12642.16 | -3.46 | -3.46 |
| Prescription of EHF | 0.35 | 0.30 | 0.39 | -12724.81 | -12572.30 | -3.49 | -3.44 |
| Intolerance to EHF | 0.40 | 0.36 | 0.44 | -12614.73 | -12669.61 | -3.46 | -3.47 |
| Does not respond to the AAF | 0.05 | 0.05 | 0.06 | -12653.29 | -12630.98 | -3.47 | -3.46 |
| Refer patient with formula intolerance for specialist consultation | 0.51 | 0.46 | 0.56 | -12642.39 | -12641.94 | -3.46 | -3.46 |
| Time to initial response assessment to diet (days) | 13.60 | 12.40 | 14.70 | -12640.58 | -12643.60 | -3.46 | -3.46 |
| Total duration of the diagnostic diet (days) | 59.40 | 50.70 | 68.20 | -12560.06 | -12726.30 | -3.44 | -3.49 |
| Medical indication of D-OFC in REAL-WORLD-OFC PRACTICE | 0.45 | 0.40 | 0.50 | -12731.34 | -12553.75 | -3.49 | -3.44 |
| Medical indication of D-OFC in GUIDELINE-OFC PRACTICE | 1.00 | 0.90 | 1.00 | -12460.62 | -12642.16 | -3.41 | -3.46 |
| D-OFC under medical supervision | 0.70 | 0.62 | 0.77 | -12646.62 | -12638.14 | -3.47 | -3.46 |
| Hospitalization for conducting the D-OFC | 0.43 | 0.35 | 0.51 | -12661.17 | -12622.96 | -3.47 | -3.46 |
| Medical recommendation for conducting the OT-OFC in REAL-WORLD-OFC PRACTICE | 0.93 | 0.92 | 0.96 | -12647.78 | -12627.54 | -3.47 | -3.46 |
| Medical recommendation for conducting the OT-OFC in GUIDELINE-OFC PRACTICE | 1.00 | 0.90 | 1.00 | -12611.23 | -12642.16 | -3.46 | -3.46 |
| OT-OFC under medical supervision | 0.65 | 0.59 | 0.69 | -12640.13 | -12643.63 | -3.46 | -3.46 |
| Hospitalization for conducting the OT-OFC | 0.36 | 0.31 | 0.41 | -12634.54 | -12649.20 | -3.46 | -3.47 |

**Abbreviations:** non-IgE-GI-CMPA, non-IgE-mediated gastrointestinal cow's milk protein allergy; EHF, extensively hydrolyzed-based formula; AAF, amino acid-based formula; OFC, oral food challenge; D-OFC, diagnostic OFC; OT-OFC, oral tolerance OFC; QALYs, quality-adjusted life years; GUIDELINE-OFC, OFC performed as recommended in guidelines; REAL-WORLD-OFC, real-world OFC practice.
